# Confounding and the healthy worker survivor effect in studies of medical radiation workers: a systematic review of methodological approaches

**DOI:** 10.4178/epih.e2026009

**Published:** 2026-02-04

**Authors:** Eun Jung Park, Kyoungyeol Yuk, Jaeho Jeong, Won Jin Lee

**Affiliations:** 1 Graduate School of Public Health, Korea University, Seoul, Korea; 2 Department of Preventive Medicine, Korea University College of Medicine, Seoul, Korea; 3 Department of Statistics, Kyungpook National University, Daegu, Korea

**Keywords:** Bias, Health personnel, Occupational exposure, Radiation, Review

## Abstract

Confounding and the healthy worker survivor effect (HWSE) represent major methodological challenges in epidemiology, particularly in studies of low-dose exposures, where effect sizes are small and risk estimates can be readily distorted by bias. This systematic review aimed to summarize the methods used to adjust for confounding and the HWSE in studies of medical radiation workers. We systematically searched PubMed and Embase for studies of medical radiation workers from inception through June 30, 2025. Studies reporting excess risk estimates for any health outcomes associated with occupational radiation exposure were included. Study selection followed the PECO (Population, Exposure, Comparator, Outcome) criteria, and data were synthesized descriptively. The review was conducted in accordance with the Preferred Reporting Items for Systematic Reviews and Meta-Analyses (PRISMA) guidelines and was registered in PROSPERO (CRD42024589851). Sixteen eligible studies from 3 countries were identified, all of which were rated as high quality. To control for confounding, regression was used in all studies, followed by stratification (62.5%) and restriction (18.8%). Age, sex, and birth year were adjusted for in all models, with smoking being the next most frequently controlled variable. To mitigate the HWSE, only a single approach, adjustment for employment characteristics, was identified, and it was applied in 3 studies (18.8%). No other approaches, including restriction or g-methods, were employed. Although confounding is generally addressed using conventional analytical approaches, the HWSE has rarely been considered in studies of medical radiation workers. More comprehensive strategies that explicitly account for the HWSE are needed to improve the validity of risk estimates, particularly in low-dose occupational studies.

## GRAPHICAL ABSTRACT

[Fig f2-epih-48-e2026009]

## Key Message

Confounding and the healthy worker survivor effect (HWSE) are major methodological challenges in epidemiology, particularly in studies involving low-dose exposure. Although confounding is generally considered to have only a limited impact in occupational epidemiology, a variety of analytical methods have nonetheless been applied to control it. In contrast, the HWSE, despite being widely recognized as a major source of bias, has rarely been mitigated in practice. More comprehensive strategies that explicitly account for the HWSE are needed to improve the validity of risk estimates.

## INTRODUCTION

Epidemiologic studies based on observational data are inherently susceptible to multiple forms of bias [1]. In occupational epidemiology, confounding constitutes a major methodological challenge because workers are often simultaneously exposed to multiple risk factors. When confounding is inadequately controlled, biased estimates of the association between exposure and disease may result [2]. In addition, the healthy worker survivor effect (HWSE) represents a particularly important source of bias, as it reflects a time-varying selection process in which healthier workers tend to remain employed longer and, as a result, accumulate greater exposure [3,4]. When healthier individuals are preferentially hired and remain employed, the observed association between exposure and disease may be underestimated, a phenomenon referred to as the healthy worker effect, which is a well-recognized issue in occupational studies [5]. These methodological concerns become especially critical in studies of workers exposed to low-dose levels, where effect sizes are typically small and risk estimates are therefore more vulnerable to bias [6].

Medical radiation workers represent the largest group of individuals occupationally exposed to man-made sources of ionizing radiation and are typically exposed at relatively low-dose levels [7]. In addition, medical radiation workers often have healthier lifestyles and greater access to healthcare services than the general population, which may contribute to a more pronounced HWSE. Previous systematic reviews of medical radiation workers have primarily focused on health outcomes, including cancer [8,9], cataract [10-12], and biomarkers of genotoxicity [13]. However, no systematic evaluation has yet examined how methodological biases, particularly confounding and the HWSE, have been identified and addressed in the estimation of health risks.

Therefore, the objective of this systematic review is to provide an overview of the methods employed to address confounding and the HWSE in studies of medical radiation workers. This review aims to inform future research and to support improvements in methodological approaches to bias assessment in low-dose epidemiologic studies.

## MATERIALS AND METHODS

### Protocol and registration

The protocol for this systematic review was developed in accordance with the Preferred Reporting Items for Systematic Reviews and Meta-Analyses (PRISMA) guidelines [14]. The protocol was registered in the PROSPERO International Prospective Register of Systematic Reviews (CRD42024589851) prior to study commencement.

### Literature search

The literature search was conducted using the PubMed and Embase databases from inception through June 30, 2025. The research question was defined according to the PECO (Population, Exposure, Comparator, Outcome) framework (Supplementary Material 1). Medical Subject Headings (MeSH) terms and text keywords included those related to the population (medical radiation workers), exposures (occupational exposure to ionizing radiation), comparisons (no or low occupational exposure), and outcomes (all diseases) (Supplementary Material 2). Search terms were combined using Boolean operators (i.e., AND, OR, and NOT). Searches were conducted without language restrictions but were limited to human studies. The same search strategy was applied to Embase using Emtree subject headings. All retrieved abstracts were reviewed to identify potentially relevant articles.

### Study selection

The inclusion criteria were as follows: (1) the study population comprised medical radiation workers, including physicians, dentists, nurses, radiological technologists, and other assistants; (2) exposures involved occupational exposure to ionizing radiation, such as X-rays; (3) comparisons comprised non-exposed or low-level occupationally exposed workers; and (4) outcomes included incidence or mortality from any disease, with effect estimates expressed as excess risks (e.g., excess relative risk, excess hazard ratio, or excess absolute risk). Inclusion was restricted to studies reporting excess risk estimates because these provide the most valid quantitative measures of radiation-related risk derived from dose assessment and help reduce information bias arising from exposure classification [15]. Exclusion criteria included experimental or interventional studies (e.g., randomized controlled trials and clinical trials), ecological and cross-sectional studies, and secondary sources such as reviews, meta-analyses, commentaries, correspondence, and news articles.

After duplicate records were removed, 2 reviewers (EJP and KY) independently screened all titles and abstracts according to the inclusion criteria. Irrelevant or ineligible studies were excluded, and full-text reviews were conducted for the remaining articles. Because this systematic review focused on methodological approaches to confounding and the HWSE, studies derived from the same cohort were considered distinct and included separately if they examined different health outcomes or employed different indices or effect measures. Final inclusion decisions were reached by consensus among all reviewers (EJP, KY, JJ, and WJL).

### Data extraction

Data extraction was performed independently by 3 reviewers (EJP, KY, and JJ) using a predefined data extraction form. For each included study, information was collected on (1) basic study characteristics (country, study name, authors, publication year, health outcomes, and risk indices), (2) methods used to control for confounding and the covariates adjusted for, and (3) approaches used to mitigate the HWSE.

Methods for confounding control were categorized as matching, restriction, stratification, and regression [5,16]. Matching pairs cases and controls by selecting comparison subjects based on 1 or more potential confounders, whereas restriction limits the study population through inclusion or exclusion criteria defined by specific values of potential confounding variables. Stratification evaluates exposure–outcome associations within strata of a potential confounder, while regression adjusts for confounders by simultaneously modeling exposure and multiple measured confounders in relation to the outcome.

All adjusted covariates were grouped into 3 main categories: demographic (e.g., age, sex, education, income, and other personal characteristics), lifestyle (e.g., smoking, alcohol consumption, body mass index, and health-related behaviors), and occupational (e.g., duration of employment, year first worked, and years since exposure). Each confounder category was counted separately when multiple categories were adjusted for within a single model.

Methods addressing the HWSE were classified as exposure lagging, restriction, adjustment for employment characteristics, and g-methods [4]. Exposure lagging excludes exposure accrued during a recent time window from cumulative exposure metrics. Restriction limits analyses to workers with sufficiently long employment duration to reduce bias resulting from early selection processes. Adjustment for employment characteristics refers to analytic approaches that incorporate employment-related variables, such as time since hire, current employment status, or time since termination, as covariates in regression models. G-methods are causal inference techniques developed to address employment status as a time-varying confounder affected by prior exposure and include the g-null test, the parametric g-formula, and g-estimation [17]. Approaches were classified as addressing the HWSE only when they were explicitly described in individual studies as strategies to mitigate the HWSE. When more than 1 method was used in a single study, all applied approaches were recorded independently. Data were grouped by country, by publication year (<2020 or ≥2020), and by health outcomes.

### Quality assessment

Quality assessment followed the recommendations of the United Nations Scientific Committee on the Effects of Atomic Radiation (UNSCEAR) 2017 to provide a comprehensive evaluation reflecting the characteristics of low-dose occupational exposure studies [15]. Eight parameters were evaluated: study participants, exposure, outcomes, design-specific bias, confounder control, statistical methods, reporting bias, and conflicts of interest. After assessing all signaling questions within each domain, a stepwise evaluation of risk-of-bias judgments and overall study quality was conducted. Quality assessment was performed independently by 2 reviewers (EJP and KY), with disagreements resolved through consensus by a third author (WJL).

### Ethics statement

Ethical approval was not required because the analysis was based exclusively on published studies.

## RESULTS

**Figure 1.**
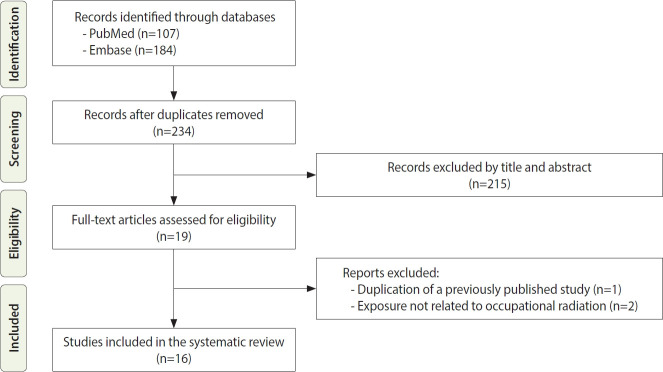
Flow diagram of the study selection process.

The initial search retrieved 291 articles (PubMed, 107; Embase, 184). After removal of 57 duplicates, 234 records were screened based on titles and abstracts. Nineteen articles were selected for full-text review, of which 3 were excluded, including 1 due to duplication of a previously published study and 2 due to non-occupational radiation exposure. Ultimately, 16 studies were included in this systematic review (Figure 1) [18-33]. All included studies were rated as high quality according to the UNSCEAR recommendations (Supplementary Material 3).

The included studies were derived from 4 cohort groups in the United States, Korea, and China, with half of the studies published after 2020 (Table 1). The U.S. Radiologic Technologists (USRT) cohort accounted for the largest proportion (56.3%), comprising 9 publications, of which 6 focused on specific cancers and 3 examined ocular diseases, including cataract, glaucoma, and macular degeneration. Korean studies investigated both cancers and circulatory diseases, whereas Chinese studies focused exclusively on solid cancers. Most studies evaluated disease incidence rather than mortality. All studies consistently adjusted for basic demographic variables, including age, sex, and birth year. Lifestyle variables (e.g., smoking, alcohol consumption, and body mass index) were considered in all USRT studies and in 2 Korean studies but were not included in Chinese studies.

Table 2 summarizes the variables adjusted for to control confounding. Demographic factors were universally controlled (100%), followed by occupational factors (93.8%) and lifestyle factors (68.8%). Among lifestyle variables, smoking was the most frequently adjusted factor (68.8%), followed by body mass index (50.0%) and alcohol consumption (37.5%). Among occupational variables, year first worked (37.5%) was most commonly adjusted, followed by duration of employment (25.0%). Other potential confounders, including marital status, education, income, diabetes, menopausal status, family history, shift work, sleep duration, and cumulative ultraviolet B (UVB) exposure, were included in only a small number of studies.

Table 3 presents the methods used to control confounding. Overall, regression (100%) and stratification (62.5%) were the most frequently applied methods, whereas restriction (18.8%) and matching (12.5%) were less commonly used. Occupational variables were primarily controlled through stratification, while demographic and lifestyle variables were typically adjusted for using regression. No meaningful differences in confounding control methods were observed across countries, publication periods, or health outcomes.

Table 4 summarizes the methods applied to mitigate the HWSE. Only 3 of the 16 studies (2 from Korea and 1 from the United States) employed any approach to address the HWSE, and these approaches were exclusively limited to adjustment for employment duration in regression models. Methods used to mitigate the HWSE did not differ substantially across countries, publication periods, or health outcomes. No studies applied alternative approaches, such as exposure lagging, restriction, or g-methods, for the explicit purpose of controlling the HWSE.

## DISCUSSION

This systematic review synthesized 16 epidemiological studies of occupational radiation exposure among medical radiation workers, with a specific focus on methodological approaches to confounding and the HWSE. We found that measured confounders were generally addressed using traditional analytical methods, such as regression and stratification. However, the HWSE was not adequately considered in most studies, despite their classification as high quality according to the UNSCEAR criteria. Because studies of low-dose exposure are particularly vulnerable to bias, future research should adopt more advanced strategies, such as g-methods, to improve the validity and precision of risk estimates.

### Confounding control

Our review showed that confounding in studies of medical radiation workers was addressed primarily at the analytical stage through regression and stratification, rather than through design-based approaches such as matching or restriction. This finding is consistent with previous systematic reviews of observational studies, which have reported regression as the most widely used method for confounding control [34,35]. One explanation is that regression offers several advantages: it allows simultaneous adjustment for multiple confounders, minimizes data loss, and is relatively efficient compared with matching, restriction, or stratification [1]. In contrast, alternative approaches have inherent limitations: matching becomes increasingly complex as the number of confounders increases, restriction reduces sample size and external validity, and stratification is practical for only a limited number of confounders [5,36].

The most frequently adjusted confounders were age, sex, and birth year, consistent with findings from other systematic reviews [8,37]. This pattern likely reflects the predominance of registry-based cohort studies, in which information is derived from administrative and occupational records but detailed individual-level data on lifestyle factors are often unavailable. Because disease occurrence is influenced by multiple determinants, access to detailed personal information is particularly important in low-dose epidemiologic studies. However, most studies face practical challenges in obtaining such information because of limitations in data collection and linkage. Nevertheless, a recent study of Korean medical radiation workers reported that radiation risk estimates adjusted only for age, sex, birth year, and employment duration did not differ substantially from estimates further adjusted for lifestyle factors, including smoking, alcohol consumption, and night shift work [38]. Similarly, a review of 43 epidemiological studies on cardiovascular effects of radiation exposure found that adjustment for lifestyle factors had little impact on reported risk estimates after controlling for age, sex, and birth year [39]. These findings suggest that adjustment for basic demographic variables may partially account for confounding by lifestyle factors. Accordingly, these variables represent not only fundamental demographic characteristics but also key confounders associated with both radiation exposure and health outcomes.

### Healthy worker survivor effect mitigation

In contrast to confounding control, methods specifically intended to address the HWSE were rarely applied. Adjustment for employment duration was the only approach identified and was used in just 3 studies (18.8%) [26,28,29]. This pattern is consistent with previous reviews indicating that employment-related adjustments are the most commonly used, but often insufficient, methods for addressing the HWSE [17]. The HWSE represents a structural source of bias arising from time-varying factors, such as health status and continued employment, that are themselves influenced by prior exposure [3]. In particular, the HWSE remains a major source of bias that has not been adequately addressed in studies of low-dose occupational radiation exposure [37]. However, in the 3 studies included in this review that adjusted for employment characteristics, time-varying confounders were treated as fixed baseline covariates. As a result, these models were unable to fully account for the HWSE, potentially leading to residual confounding or even bias amplification [40].

Although all included studies were rated as high quality according to the 2017 UNSCEAR recommendations, this assessment did not sufficiently capture bias arising from the HWSE. Given that all included studies met high-quality criteria, bias control is likely to be even less adequate in studies not captured by this review. Other commonly used quality assessment tools, such as the Newcastle–Ottawa Scale [41] and ROBINS-I [42], exhibit similar limitations. This suggests that even comprehensive assessment frameworks may fail to adequately identify key context-specific biases, such as the HWSE, when these are not explicitly defined as separate evaluation domains [43].

All included studies applied exposure lagging, but this was intended to account for disease latency rather than to mitigate the HWSE. Exposure lagging is typically anchored to disease onset, reflecting the interval between exposure and diagnosis. In contrast, mitigation of the HWSE requires anchoring on employment cessation, because the bias arises from the preferential retention of healthier workers. Therefore, although the lagging approaches used in these studies may have partially reduced the HWSE, none of the studies described exposure lagging as a strategy to address the HWSE, nor did they explicitly justify its use for that purpose.

Notably, no study applied advanced causal inference approaches, such as g-methods, to address the HWSE. This likely reflects both technical and data-related challenges associated with these methods, which require detailed longitudinal information, including repeated measurements of time-varying exposures, employment status, and health status, as well as advanced statistical expertise [44,45]. Most studies of medical radiation workers rely on retrospective cohort designs with limited information on health status or employment transitions, which constrains the feasibility of such approaches. Although g-estimation has recently been introduced into occupational epidemiology [46], its application to radiation worker studies remains limited. Recent analyses of Korean medical radiation workers using g-estimation have demonstrated that failure to adjust for the HWSE can lead to substantial underestimation, by approximately 30–50%, of radiation-related cancer incidence and mortality risks among males [47]. Therefore, application of advanced causal inference methods is essential for adequately addressing the HWSE and for obtaining unbiased risk estimates in low-dose occupational studies.

## CONCLUSION

This systematic review provides methodological insights into how confounding and the HWSE have been addressed in studies of medical radiation workers. Although confounding is generally considered to have a limited impact in occupational epidemiology, a range of analytical methods has nonetheless been applied to control for it. In contrast, the HWSE, despite being widely recognized as a major source of bias, has rarely been addressed in practice. Therefore, future low-dose epidemiologic studies should place greater emphasis on explicitly accounting for the HWSE to improve the validity of risk estimates.

## Figures and Tables

**Figure f2-epih-48-e2026009:**
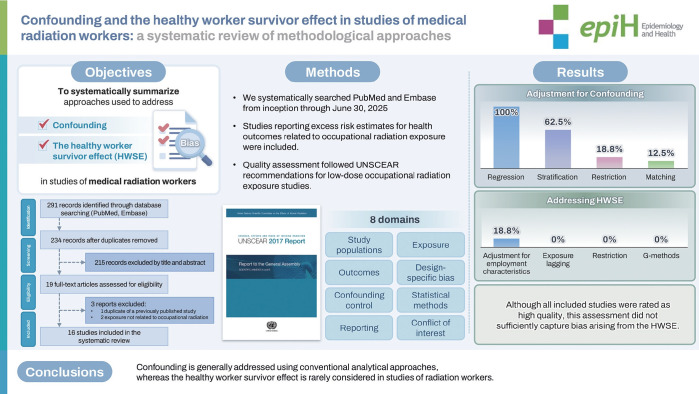


**Table 1.** Methods used to control confounding and to mitigate the healthy worker survivor effect in studies of medical radiation workers

Study	Confounding control (methods and variables)	Mitigation of the healthy worker survivor effect (methods and variables)	Outcomes	Indices
USA/U. S. Radiologic Technologists (USRT)				
Linet et al., 2020 [18]	Regression (age, sex, birth year, smoking)	-	Hematopoietic malignancy	Mortality
	Stratification (sex, birth year, smoking, year first worked, ever worked with fluoroscopically guided procedures, ever worked with nuclear medicine procedures)			
Little et al., 2020 [19]	Regression (age, sex, birth year, diabetes, BMI, smoking, cumulative UVB radiation exposure, year of follow up)	-	Cataract	Incidence
	Stratification (age, sex, birth year, racial/ethnic group, diabetes, BMI, smoking, cumulative UVB radiation exposure)			
Velazquez-Kronen et al., 2020 [20]	Regression (age, sex, birth year, smoking)	-	Lung cancer	Mortality
	Stratification (age, sex, birth year, smoking, ever worked with fluoroscopically guided procedures, ever worked with nuclear medicine procedures, year first worked)			
Little et al., 2018 [21]	Regression (age, sex, birth year, diabetes, BMI, smoking, cumulative UVB radiation exposure)	-	Cataract	Incidence
	Stratification (age, sex, birth year, racial/ethnic group, diabetes, BMI, smoking, cumulative UVB radiation exposure, risk by age at exposure, risk by time since exposure)			
Little et al., 2018 [22]	Regression (age, sex, birth year, race, BMI, diabetes, smoking)	-	Glaucoma and macular degeneration	Incidence
Kitahara et al., 2018 [23]	Regression (age, sex, birth year, smoking, alcohol, BMI)	-	Thyroid cancer	Incidence
	Stratification (sex, birth year, year first worked, ever worked with fluoroscopically guided procedures, ever worked with nuclear medicine procedures)			
Kitahara et al., 2017 [24]	Regression (age, sex, birth year, race, marital status, height, BMI, smoking, alcohol, no. of live births, menopausal status, hormone therapy uses, medical history of asthma, medical history of diabetes)	-	Brain, CNS	Mortality
	Stratification (sex, birth year, year first worked, work with fluoroscopically guided procedures)			
Preston et al., 2016 [25]	Regression (age, birth year, race, marital status, smoking, BMI, alcohol, no. of live births, menopause status, use of hormone replacement therapy, family history of breast cancer)	-	Breast cancer	Incidence and mortality
	Restriction (sex)			
	Stratification (birth year, year first worked)			
Lee et al., 2015 [26]	Regression (age, sex, birth year, calendar year, education, income, smoking, alcohol, BMI, exercise, eye color, hair color, skin complexion, skin reaction to strong sunlight, UVR score, sunburn history, dental X-rays)	Adjustment for employment characteristics (duration of employment)	Basal cell carcinoma	Incidence
	Restriction (race)			
	Stratification (age during exposure, years since exposure, calendar year of exposure)			
USA/Million Person Study				
Boice et al., 2023 [27]	Regression (age, sex, birth year, occupational category)	-	Lung cancer, IHD, leukemia, etc.	Mortality
Korea/Diagnostic medical radiation workers				
Bang et al., 2023 [28]	Regression (age, birth year, smoking, alcohol, year of employment duration, sleep duration, shift work)	Adjustment for employment characteristics (duration of employment)	Circulatory disease	Mortality
	Restriction (sex)			
Lee et al., 2021 [29]	Regression (age, sex, birth year, duration of employment)	Adjustment for employment characteristics (duration of employment)	All cancer and specific cancers	Incidence
Cha et al., 2020 [30]	Regression (age, sex, birth year, smoking, family history of hypertension, family history of stroke, family history of heart, alcohol, BMI, blood pressure, blood glucose, total cholesterol)	-	Circulatory disease	Incidence
	Stratification (age, sex, birth year, year of starting work, total years worked)			
Lee et al., 2019 [31]	Regression (age, sex, calendar year)	-	Thyroid cancer	Incidence
	Stratification (sex, birth year, year of entry, duration of employment, job title, age at baseline, type of medical facility)			
China/Chinese medical X-ray workers				
Gu et al., 2023 [32]	Matching (the same period of employment, hospital)	-	Specific solid cancers	Incidence
	Regression (age, sex, birth year)			
Sun et al., 2016 [33]	Matching (the same period of employment, hospital)	-	Solid cancers	Incidence
	Regression (age, sex, birth year)			

BMI, body mass index; CNS, central nervous system; IHD, ischemic heart disease; UVB, ultraviolet B; UVR, solar ultraviolet radiation.

**Table 2.** Confounding variables adjusted for in studies of medical radiation workers

Variables	n (%)
Demographic factors	
Age	16 (100)
Sex	16 (100)
Birth year	16 (100)
Race	6 (37.5)
Calendar year	2 (12.5)
Marital status	2 (12.5)
Education	1 (6.3)
Income	1 (6.3)
Eye color	1 (6.3)
Height	1 (6.3)
Skin complexion	1 (6.3)
Personal radiation examinations	1 (6.3)
Lifestyle factors	
Smoking	11 (68.8)
Body mass index	8 (50.0)
Alcohol consumption	6 (37.5)
Diabetes	3 (18.8)
Family history	2 (12.5)
Menopausal hormone therapy uses	2 (12.5)
Menopausal status	2 (12.5)
No. of live births	2 (12.5)
Exercise	1 (6.3)
Blood pressure	1 (6.3)
Blood glucose	1 (6.3)
Shift work	1 (6.3)
Sleep duration	1 (6.3)
Total cholesterol	1 (6.3)
Occupational factors	
Year first worked	6 (37.5)
Duration of employment	4 (25.0)
Ever worked with fluoroscopically guided or nuclear medicine procedures	4 (25.0)
Hospital	2 (12.5)
Same period of employment	2 (12.5)
Years since exposure	2 (12.5)
Calendar year of exposure	1 (6.3)
Job title	1 (6.3)
Occupational category	1 (6.3)
Year of follow-up	1 (6.3)
Type of medical facility	1 (6.3)
Other factors	
Ultraviolet B	2 (12.5)
Solar ultraviolet radiation	1 (6.3)
Dental X-ray	1 (6.3)

**Table 3.** Methods used to adjust for confounding variables by study characteristics

Variables	Regression	Stratification	Restriction	Matching
All (n=16)	16 (100)	10 (62.5)	3 (18.8)	2 (12.5)
Variable category				
Demographic (n=16)^1^	16 (100)	9 (56.3)	3 (18.8)	0 (0)
Occupational (n=15)^2^	4 (26.7)	9 (60.0)	0 (0)	2 (13.3)
Lifestyle (n=11)^3^	11 (100)	4 (36.4)	0 (0)	0 (0)
Country				
USA (n=10)	10 (100)	8 (80.0)	2 (20.0)	0 (0)
Korea (n=4)	4 (100)	2 (50.0)	1 (25.0)	0 (0)
China (n=2)	2 (100)	0 (0)	0 (0)	2 (100)
Publication year				
<2020 (n=8)	8 (100)	6 (75.5)	2 (25.0)	1 (12.5)
≥2020 (n=8)	8 (100)	4 (50.0)	1 (12.5)	1 (12.5)
Health outcomes				
Cancer (n=10)	10 (100)	7 (70.0)	2 (20.0)	2 (20.0)
Non cancer (n=5)	5 (100)	3 (60.0)	1 (20.0)	0 (0)
Cancer and non-cancer (n=1)	1 (100)	0 (0)	0 (0)	0 (0)

Values are presented as number (%).

1 Demographic variables include age, sex, education, income, and other personal characteristics.

2 Ocupational variables include duration of employment, year first worked, and years since exposure.

3 Lifestyle variables include smoking, alcohol consumption, body mass index, and other health-related behaviors.

**Table 4.** Methods to mitigate the healthy worker survivor effect by study characteristics

Variables	Adjustment for employment characteristics	Restriction	Exposure lagging	G-methods
All (n=16)	3 (18.8)	0 (0)	0 (0)	0 (0)
Country				
USA (n=10)	1 (10.0)	0 (0)	0 (0)	0 (0)
Korea (n=4)	2 (50.0)	0 (0)	0 (0)	0 (0)
China (n=2)	0 (0)	0 (0)	0 (0)	0 (0)
Publication year				
<2020 (n=8)	1 (12.5)	0 (0)	0 (0)	0 (0)
≥2020 (n=8)	2 (25.0)	0 (0)	0 (0)	0 (0)
Health outcomes				
Cancer (n=10)	2 (20.0)	0 (0)	0 (0)	0 (0)
Non cancer (n=5)	1 (20.0)	0 (0)	0 (0)	0 (0)
Cancer and non-cancer (n=1)	0 (0)	0 (0)	0 (0)	0 (0)

Values are presented as number (%).

## Supplementary materials

Supplementary Material 1. PECO statement

Supplementary Material 2. Search queries

Supplementary Material 3. Quality assessment by domain for the included studies of medical radiation workers
