# Supplementary material for: Confounding and the healthy worker survivor effect in studies of medical radiation workers: a systematic review of methodological approaches
Source: Epidemiol Health. 2026 Feb 4;48:e2026009. doi: 10.4178/epih.e2026009 (PMC13033441; doi:10.4178/epih.e2026009)
Supplement: Supplementary Material 1. — PECO statement [file epih-48-e2026009-Supplementary-1.docx]

Supplementary Material 1. PECO statement

| PECO element | Evidence stream | | Articles or features included | | Articles or features excluded |
| --- | --- | --- | --- | --- | --- |
| Population | Human | | • Medical worker  • All life stages | | - |
| Exposure | Human | | • Ionizing radiation exposure (e.g., Gamma, X-ray)  • Occupational exposure  • Quantitative in units of equivalent or  effective dose (e.g., mSv, Sv) or absorbed dose (e.g., mGy, Gy) | | - |
| Comparison | Human | | • A comparison population [no occupational exposure or lower occupational exposure]  • Effect measurements reported as ERR, EHR, EAR | | - |
| Outcome | Human | | • Endpoints: All diseases (mortality or incidence) | | - |
| General considerations | | • Reports primary observational study (e.g., cohort or case-control)  • Full text available | | • Experimental or interventional studies (e.g., randomized controlled trials, clinical trials)  • Ecological and cross-sectional studies  • Secondary or non-original sources (e.g., reviews, meta-analyses, editorials, correspondence, proceedings, news articles) | |
