# Supplementary material for: Confounding and the healthy worker survivor effect in studies of medical radiation workers: a systematic review of methodological approaches
Source: Epidemiol Health. 2026 Feb 4;48:e2026009. doi: 10.4178/epih.e2026009 (PMC13033441; doi:10.4178/epih.e2026009)
Supplement: Supplementary Material 2. — Search queries [file epih-48-e2026009-Supplementary-2.docx]

Supplementary Material 2. Search queries

| PubMed | |
| --- | --- |
| Population | (“Health Personnel”[Mesh] OR “Radiologic technologist*”[tiab] OR “Radiologist*”[tiab] OR “Cardiologist*”[tiab] OR “Surgeon*”[tiab] OR “X-ray worker*”[tiab] OR “Doctor*”[tiab] OR “Physician*”[tiab] OR “Radiotherapy department*”[tiab] OR “Health Personnel”[tiab] OR “Medical worke*”[tiab] OR “Medical radiation worker*”[tiab] OR “Medical Staff*”[tiab] OR “Health worker*”[tiab] OR “radiological technologist*”[tiab]) |
| Exposure | (“Radiation, Ionizing”[Mesh] OR “Ionizing Radiat*”[tiab] OR “Ionising Radiat*”[tiab] OR “Radiation Exposure”[tiab] OR “Radiation-Induced”[tiab] OR “Radiation Induced”[tiab] OR “Radiation-Associated”[tiab] OR “Radiation Associated”[tiab] OR "X-Rays"[Mesh] OR "X-Ray*"[tiab] OR “Occupations”[Mesh] OR “Occupational Exposure”[Mesh:NoExp] OR “Occupat*”[tiab] OR “Occupational Exposure”[tiab] OR “Occupational Radiation Exposure”[tiab]) |
| Comparison | ("Excess Relative Risk*"[tiab] OR "Excess Absolute Risk*"[tiab] OR "Excess Hazard Ratio*”[tiab] OR “ERR”[tiab] OR “EAR”[tiab] OR “EHR”[tiab]) |
| Outcome | (“Neoplasms”[Mesh] OR “Neoplasms, Radiation-Induced”[Mesh] OR “Leukemia, Radiation-Induced”[Mesh] OR “Neoplasm*”[tiab] OR “Cancer*”[tiab] OR “Leukemia*”[tiab] OR “Malignant Tumor*”[tiab] OR “Cardiovascular Diseases”[Mesh] OR “Myocardial Infarction”[Mesh] OR “Stroke”[Mesh] OR “Hypertension”[Mesh] OR “Heart Failure”[Mesh] OR “Respiratory Tract Diseases”[Mesh] OR “Pulmonary Fibrosis”[Mesh] OR “Thyroid Diseases”[Mesh] OR “Cataract”[Mesh] OR “Liver Diseases”[Mesh] OR “Kidney Diseases”[Mesh] OR “Neurologic Manifestations”[Mesh] OR “Neurodegenerative Diseases”[Mesh] OR “Non-neoplastic disease*”[tiab] OR “Cardiovascular disease*”[tiab] OR “CVD”[tiab] OR “Stroke”[tiab] OR “Myocardial infarction”[tiab] OR “Heart disease*”[tiab] OR “Respiratory disease*”[tiab] OR “Lung disease*”[tiab] OR “Pulmonary”[tiab] OR “Thyroid disease*”[tiab] OR “Cataract*”[tiab] OR “Neurodegenerative”[tiab] OR “Parkinson*”[tiab] OR “Dementia”[tiab] OR “Alzheimer*”[tiab]) |
| Final search query | (#1 AND #2 AND #3 AND #4) NOT (Review[pt] OR “Review Literature as Topic”[Mesh]) AND Humans[Mesh] AND ("1000/01/01" : "2025/06/30"). |

(Continued)

| Embase | |
| --- | --- |
| Population | (‘health care personnel’)/exp OR (‘Radiologic technologist*’ OR ‘Radiologist*’ OR ‘Cardiologist*’ OR ‘Surgeon*’ OR ‘X-ray worker*’ OR ‘Doctor*’ OR ‘Physician*’ OR ‘Radiotherapy department*’ OR ‘Health Personnel’ OR ‘Medical worke*’ OR ‘Medical radiation worker*’ OR ‘Medical Staff*’ OR ‘Health worker*’ OR ‘radiological technologist*’):ab,ti |
| Exposure | (‘Ionizing Radiation’ OR ‘X ray’ OR ‘Occupation’ OR ‘Occupational exposure’)/exp OR (**‘**Ionizing Radiat*’ OR ‘Ionising Radiat*’ OR ‘Radiation Exposure’ OR ‘Radiation-Induced’ OR ‘Radiation Induced’ OR ‘Radiation-Associated’ OR ‘Radiation Associated’ OR ‘X-Ray*’ OR ‘Occupat*’ OR ‘Occupational Exposure’ OR ‘Occupational Radiation Exposure’):ab,ti |
| Comparison | (‘Excess Relative Risk*’ OR ‘Excess Absolute Risk*’ OR ‘Excess Hazard Ratio*’ OR ‘ERR’ OR ‘EAR’ OR ‘EHR’):ab,ti |
| Outcome | (‘Neoplasm' OR 'radiation induced neoplasm' OR 'radiation induced leukemia' OR 'cardiovascular disease' OR 'myocardial infarction' OR ‘stroke’ OR ‘hypertension’ OR 'heart failure' OR 'respiratory tract disease’ OR 'pulmonary fibrosis' OR 'thyroid disease' OR ‘cataract’ OR 'liver disease' OR 'kidney disease' OR 'neurologic manifestation' OR 'neurodegenerative disease')/exp OR (‘neoplasm*’ OR 'neoplasms, radiation-induced' OR 'leukemia, radiation-induced' OR ‘cancer*’ OR ‘leukemia*’ OR 'malignant tumor*' OR 'cardiovascular disease*' OR ‘cvd’ OR ‘stroke’ OR 'myocardial infarction' OR 'heart disease*' OR 'respiratory disease*' OR 'lung disease*' OR ‘pulmonary’ OR 'thyroid disease*' OR ‘cataract*’ OR ‘neurodegenerative’ OR ‘parkinson*’ OR ‘dementia’ OR ‘alzheimer*’ OR 'non neoplastic disease*'):ab,ti |
| Final search query | \| (#1 AND #2 AND #3 AND #4) NOT ('conference abstract':it OR 'conference review':it OR 'review':it) AND [humans]/lim AND [01-01-1000]/sd NOT [01-07-2025]/sd \| \| --- \| |
