# Supplementary material for: Confounding and the healthy worker survivor effect in studies of medical radiation workers: a systematic review of methodological approaches
Source: Epidemiol Health. 2026 Feb 4;48:e2026009. doi: 10.4178/epih.e2026009 (PMC13033441; doi:10.4178/epih.e2026009)
Supplement: Supplementary Material 3. — Quality assessment by domain for the included studies of medical radiation workers [file epih-48-e2026009-Supplementary-3.docx]

Supplementary Material 3. Quality assessment by domain for the included studies of medical radiation workers

| Authors, Year  [reference] | Risk of bias domain | | | | | | | | Overall quality assessment |
| --- | --- | --- | --- | --- | --- | --- | --- | --- | --- |
|  | Study participants  (selection bias) | Exposure  (performance bias) | Outcomes  (detection bias) | Design-specific bias  (attrition bias, other biases) | Confounder  Control  (other biases) | Statistical  Methods  (other biases) | Reporting  (other biases) | Conflict  of interesting |  |
| Linet et al.,  2020 [18] | Low | Low | Low | Low | Low | Low | Low | Low | High |
| Little et al.,  2020 [19] | Low | Low | Low | Low | Low | Low | Low | Low | High |
| Velazquez-Kronen et al., 2020 [20] | Low | Low | Low | Low | Low | Low | Low | Low | High |
| Little et al.,  2018 [21] | Low | Low | Low | Low | Low | Low | Low | Low | High |
| Little et al.,  2018 [22] | Low | Low | Low | Low | Low | Low | Low | Low | High |
| Kitahara et al., 2018 [23] | Low | Low | Low | Low | Low | Low | Low | Low | High |
| Kitahara et al., 2017 [24] | Low | Low | Low | Low | Low | Low | Low | NI | High |
| Preston et al., 2016 [25] | Low | Low | Low | Low | Low | Low | Low | Low | High |
| Lee et al.,  2015 [26] | Low | Low | Low | Low | Low | Low | Low | Low | High |
| Boice et al.,  2022 [27] | Low | Low | Low | Low | Low | Low | Low | Low | High |

(Continued)

| Authors, Year  [reference] | Risk of bias domain | | | | | | | | Overall quality assessment |
| --- | --- | --- | --- | --- | --- | --- | --- | --- | --- |
|  | Study participants  (selection bias) | Exposure  (performance bias) | Outcomes  (detection bias) | Design-specific bias  (attrition bias, other biases) | Confounder  Control  (other biases) | Statistical  Methods  (other biases) | Reporting  (other biases) | Conflict  of interesting |  |
| Bang et al.,  2023 [28] | Low | Low | Low | Low | Low | Low | Low | Low | High |
| Lee et al.,  2021 [29] | Low | Low | Low | Low | Low | Low | Low | Low | High |
| Cha et al.,  2020 [30] | Low | Low | Low | Low | Low | Low | Low | Low | High |
| Lee et al.,  2019 [31] | Low | Low | Low | Low | Low | Low | Low | Low | High |
| Gu et al.,  2023 [32] | Low | Low | Low | Low | Low | Low | Low | Low | High |
| Sun et al.,  2016 [33] | Low | Low | Low | Low | Low | Low | Low | NI | High |
